# Effective Identification of Maternal Malignancies in Pregnancies Undergoing Noninvasive Prenatal Testing 

**DOI:** 10.3389/fgene.2022.802865

**Published:** 2022-02-10

**Authors:** Jia Li, Jia Ju, Qiang Zhao, Weiqiang Liu, Yuying Yuan, Qiang Liu, Lijun Zhou, Yuan Han, Wen Yuan, Yonghua Huang, Yingjun Xie, Zhihua Li, Jingsi Chen, Shuyu Huang, Rufang Chen, Wei Li, Meihua Tan, Danchen Wang, Si Zhou, Jian Zhang, Fanwei Zeng, Nan Yu, Fengxia Su, Min Chen, Yunsheng Ge, Yanming Huang, Xin Jin

**Affiliations:** ^1^ BGI Genomics, BGI-Shenzhen, Shenzhen, China; ^2^ Hebei Industrial Technology Research Institute of Genomics in Maternal & Child Health, Shijiazhuang BGI Genomics Co., Ltd., Shijiazhuang, China; ^3^ BGI-Shenzhen, Shenzhen, China; ^4^ BGI Education Center, University of Chinese Academy of Sciences, Shenzhen, China; ^5^ Department of Obstetrics and Gynecology, Jiangmen Central Hospital, Jiangmen, China; ^6^ Reproductive Medicine Center, Jiangmen Central Hospital, Jiangmen, China; ^7^ Central Lab, Longgang District Maternity & Child Healthcare Hospital of Shenzhen City, Shenzhen, China; ^8^ BGI-Wuhan, BGI-Shenzhen, Wuhan, China; ^9^ Department of Obstetrics and Gynecology, Key Laboratory for Major Obstetric Diseases of Guangdong Province, The Third Affiliated Hospital of Guangzhou Medical University, Guangzhou, China; ^10^ Department of Prenatal Diagnosis and Fetal Medical, Third Affiliated Hospital of Guangzhou Medical University, Guangzhou, China; ^11^ The Department of Obstetrics, Foshan First People’s Hospital, Foshan, China; ^12^ School of Basic Medicine, Qingdao University, Qingdao, China; ^13^ College of Life Sciences, University of Chinese Academy of Sciences, Beijing, China; ^14^ Department of Prenatal Diagnosis Center, Women and Children’s Hospital, School of Medicine, Xiamen University, Xiamen, Fujian province, China; ^15^ Clinical Experimental Center, Jiangmen Central Hospital, Jiangmen, Guangdong province, China; ^16^ School of Medicine, South China University of Technology, Guangzhou, China

**Keywords:** cell-free DNA, maternal malignancy, non-invasive prediction, random forest, classifier

## Abstract

**Background:** The existence of maternal malignancy may cause false-positive results or failed tests of NIPT. Though recent studies have shown multiple chromosomal aneuploidies (MCA) are associated with malignancy, there is still no effective solution to identify maternal cancer patients from pregnant women with MCA results using NIPT. We aimed to develop a new method to effectively detect maternal cancer in pregnant women with MCA results using NIPT and a random forest classifier to identify the tissue origin of common maternal cancer types.

**Methods:** For examination, 496 participants with MCA results via NIPT were enrolled from January 2016 to June 2019 at BGI. Cancer and non-cancer participants were confirmed through the clinical follow-up. The cohort comprising 42 maternal cancer cases and 294 non-cancer cases enrolled from January 2016 to December 2017 was utilized to develop a method named mean of the top five chromosome z scores (MTOP5Zscores). The remaining 160 participants enrolled from January 2018 to June 2019 were used to validate the performance of MTOP5Zscores. We established a random forest model to classify three common cancer types using normalized Pearson correlation coefficient (NPCC) values, z scores of 22 chromosomes, and seven plasma tumor markers (PTMs) as predictor variables.

**Results:** 62 maternal cancer cases were confirmed with breast cancer, liver cancer, and lymphoma, the most common cancer types. MTOP5Zscores showed a sensitivity of 85% (95% confidence interval (CI), 62.11–96.79%) and specificity of 80% (95% CI, 72.41–88.28%) in the detection of maternal cancer among pregnant women with MCA results. The sensitivity of the classifier was 93.33, 66.67, and 50%, while specificity was 66.67, 90, and 97.06%, and positive predictive value (PPV) was 60.87, 72.73, and 80% for the prediction of breast cancer, liver cancer, and lymphoma, respectively.

**Conclusion:** This study presents a solution to identify maternal cancer patients from pregnant women with MCA results using NIPT, indicating it as a value-added application of NIPT in the detection of maternal malignancies in addition to screening for fetal aneuploidies with no extra cost.

## Introduction

Noninvasive prenatal testing (NIPT) first became commercially available to screen for fetal trisomy-21 in 2011 and went global with a rapid speed (Agarwal et al., 2013). The high sensitivity and specificity for NIPT to detect fetal trisomy-21, -18, and -13 are now well recognized and widely applied in clinical practice (Chen et al., 2011; Benn et al., 2013), but as more tests are performed globally, the issues related to false positives and inconclusive test results are coming to the foreground. The discordant results between cell-free DNA and fetal karyotype could be attributed to various factors, such as confined placental mosaics (Lau et al., 2013), co-twin demise (Curnow et al., 2015), maternal chromosomal mosaics (Bianchi et al., 2015b), and maternal malignancy (Bianchi et al., 2015a; Amant et al., 2015; Hartwig et al., 2017).

Maternal malignancy is relatively rare in pregnancy, with an incidence rate of 1 in 1,000 pregnancies (Pavlidis, 2002). Thereinto, breast cancer, melanoma, cervical cancer, and Hodgkin’s disease are the most common cancer types during pregnancy (Albright and Wenstrom, 2016). Incidental discovery of maternal cancer has been repeatedly reported within failed NIPTs due to multiple chromosomal aneuploidies (MCA) (Osborne et al., 2013; Bianchi et al., 2015a; Amant et al., 2015; Dharajiya et al., 2018). In 2013, Osborne et al. reported the first case of maternal malignancy with discordant NIPT results. A pregnant woman had aneuploidies of chromosome 13 and 18 found via an NIPT and was subsequently diagnosed with metastatic disease of small-cell carcinoma of vaginal origin (Osborne et al., 2013). In 2015, Bianchi discovered 10 maternal cancer cases from 125,426 pregnancies based on aneuploidies involving chromosomes 13, 18, 21, X, or Y via NIPT. Eight cancer cases showed nonspecific copy-number gains and losses across multiple chromosomes (Bianchi et al., 2015a). In 2017, Dharajiya reported 18 malignant maternal malignancies in 43 non-reportable NIPT cases with altered genomic profiles (Dharajiya et al., 2018). These studies suggest that aneuploidies involving multiple chromosomes are associated with the development of maternal cancer.

Our previous study has proposed a method named cancer detection pipeline which performs genomic profiling for copy-number variations (CNVs) of plasma DNA to identify incidental maternal malignancies (Ji et al., 2019). Nevertheless, there are multiple limitations in the previous study, such as the complexity of the bioinformatics algorithm, lack of independent validation, and ineffectiveness of tumor origin identification for suspicious cases. Here, we present a retrospective study involving 496 participants with MCA results from NIPT. The purpose of this study was to refine the performance of NIPT in the identification of incidental maternal malignancies by developing bioinformatics algorithms and a tissue origin classifier for common maternal cancer types.

## Methods

### Sequencing and Bioinformatics Analysis

Five milliliters of maternal peripheral blood were collected in a Streck Cell-Free DNA BCT ^®^ blood collection tube (Streck, La Vista, Nebraska, United States) and were processed within 4 days of collection. Details of the NIPT method, also called the noninvasive fetal trisomy test (NIFTY), have been published previously (Lau et al., 2012). In brief, plasma was separated by sequential centrifugations of the blood sample at 1600 g at 4°C for 10 min. Cell-free DNA was extracted from plasma and subjected to library construction. The quantity and quality of the library were examined by real-time polymerase chain reaction and size distribution analysis. Only the qualified libraries were sequenced, and the data generated were analyzed using bioinformatics algorithms to detect fetal chromosomal aneuploidy and large deletions and duplications as previously described (Lau et al., 2012; Article, 2013). MCA was defined as at least two chromosomes having absolute z-scores >3.0.

### Participants

We retrospectively enrolled participants with MCA records between January 2016 and June 2019 at BGI-Shenzhen and BGI-Wuhan. Participants were retrospectively interviewed by physicians every 6 months through telephone and online questionnaires (Supplementary Method 1). Clinical information regarding patients’ medical information was obtained from patients or their clinicians with a questionnaire (Supplementary Method 2). Non-cancer participants with positive MCA results were regarded as participants but no cancer was identified at the last time of follow-up.

### Development of the Maternal Cancer Predictor

The mean of the top five chromosome z scores (MTOP5Zscores) represented the mean of the top five chromosomes with the largest absolute z scores except for chromosomes Y and 19. In our cohort, the frequencies of chr19 deletion and chr19 amplification were 53.92 and 21.89%, respectively, in non-cancer participants (Supplementary Figure S1). Therefore, chr19 was excluded in the calculation of MTOP5Zscores. MTOP5Zscores were retrospectively computed for 496 participants. The participants enrolled from January 2016 to December 2017 were utilized as a training set to develop the MTOP5Zscore method and determine the optimal cutoff value; the training set comprised 42 maternal cancers cases and 294 non-cancer cases. The participants enrolled from January 2018 to June 2019 were used to independently validate the performance of MTOP5Zscores. The validation set comprised 20 maternal cancer cases and 140 non-cancer participants. The R package of pROC was used to compute sensitivities and specificities, build receiver operating characteristic (ROC) curves, and calculate the area under the curve (AUC) values for MTOP5Zscores (Robin et al., 2011).

### Calculation and Normalization of Read Coverage for Genes

The raw data in the fastq format of maternal liver cancer, breast cancer, and lymphoma cases were mapped to a human reference genome (hg19) by using the Burrow–Wheeler Aligner (BWA) tool (Li and Durbin, 2009). Reads with mapping quality score below 30 and polymerase chain reaction (PCR) duplicates were removed by using the Picard tool. The bam file was used to predict copy-number variations by using HMMcopy with 100 kb resolution (Lai and Ha, 2016). For all the mapped reads, we recorded their start position. Reverse-mapped reads had their start position adjusted for their length by adding their length minus one base pair (bp) to their first position on the genome. In order to enhance the nucleosome signal, the read start position was extended 167bp; then, the central 61bp (53–113) of 167-bp cfDNA fragments were used to calculate the read depth. Read depth of each site was normalized by dividing read depth by median log2 scaled copy-number variation ratio with 100 kb resolution. For each transcript in the RefSeq database, accumulative read depths were calculated in 1,000 flanking regions around the transcriptional start site (TSS) and then normalized using the read depths per kilobase per million mapped reads (RDPKM). For the genes that have more than one transcript, the mean RDPKM value was calculated. The genes with average RDPKM <100 were eliminated from this study.

### Calculation of Normalized Pearson’s Correlation Coefficient

Several studies demonstrate that gene expression levels have a negative correlation with the accumulative read depths across the TSS region (Ulz et al., 2016; Guo et al., 2020). In our study, we took the gene expression values of breast invasive carcinoma (BRCA) (Cancer and Atlas, 2012), diffuse large B-cell lymphoma (DLBC, https://tcga-data.nci.nih.gov/tcga/), and liver hepatocellular carcinoma (LIHC) (Ally et al., 2017) from The Cancer Genome Atlas (TCGA) database as a reference. We calculated the mean fragments per kilobase of gene per million mapped reads (FPKM) values of each gene in 429 LIHC samples, 885 BRCA samples, and 48 DLBC samples, and the genes with log2 (mean FPKM) < 0.1 were removed from the study. For each maternal cancer sample, RDPKM values in the TSS region, and Pearson’s correlations between RDPKM and the mean FPKM values of LIHC, BRCA, and DLBC were calculated separately with the mean of 14,589 genes. The coefficient of the Pearson’s correlation was divided by the sum of the three coefficients of BRAC, DLBC, and LIHC for each maternal cancer sample.

$$Normalized\;Pearson's\;correlation\;coefficient\left(NPCC\right)R_{i}=\frac{R_{i}}{\sum R_{i}}$$

i∈(BRAC,DLBC,LIHC),

where *R*_*i*_ is the coefficient of Pearson’s correlation between the RDPKM and the mean FPKM value of cancer type i.

### Analysis of Plasma Tumor Markers

PTMs test has been widely implemented in clinical settings to increase diagnostic accuracy in several cancer indications as well as to monitor disease progression (Borrebaeck, 2017). Plasma tumor markers (PTMs) were retrospectively tested in 56 maternal cancer cases and 451 non-cancer cases using the remaining plasma after NIPTs. A microarray enzyme-linked immunoassay test was performed to detect the concentration of plasma tumor markers, following the manufacturer’s instruction (Beijing BGI-GBI Biotech Co., Ltd., Beijing, China). The tumor markers include CA15-3 (carbohydrate antigen 15–3), AFP (alpha-fetoprotein), CEA (carcinoembryonic antigen), CA19-9 (carbohydrate antigen 19–9), CA125 (carbohydrate antigen 125), CA72-4 (cancer antigen 72–4), and CYFRA21-1 (human cytokeratin fragment antigen 21–1). The respective cutoff values recommended by the assay manufacturer were 28 U/ml, 500 ng/ml, 5 ng/ml, 37 U/ml, 36 U/ml, 3.3 ng/ml, 1.2 ng/ml for CA15-3, AFP, CEA, CA19-9, CA125, CYFRA21-1, and SCC, respectively.

### Development of Tumor Tissue Origin Classifier

The tumor tissue origin classifier was built by the R package randomForest with ntree = 500 and default mtry values (Breiman, 2001). The three NPCC values, z scores of 22 chromosomes and seven PTMs were set as predictor variables and cancer types comprising breast cancer, liver cancer, lymphoma, and gastric cancer as response variables. Leave-one-out cross-validation was performed to evaluate the accuracy and robustness of the tumor tissue origin classifier. For each iteration, the random forest classifier used one sample as the test set and the remaining samples as the training set and generated the probability for each cancer type and predicted the cancer type of the test sample. The ROC curves of the classifier were plotted, and the AUC values were computed by the python package sklearn using probabilities predicted by the random forest classifier.

### Statistical Analyses

Data were presented as the mean ± SD (standard deviation). MTOP5Zscores were compared between cancer and non-cancer groups using the Wilcoxon sum rank test in R. The Kruskal–Wallis test was used to compare the differences in MTOP5Zscores in cancer patients at different stages. The Kaplan–Meier method was used to plot diagnostic curves, and the log-rank test was utilized to compare the difference in non-cancer rates between different groups. *p* < 0.05 was predefined to indicate a statistically significant difference.

## Results

### Overview of Maternal Cancer Cases Identified in 496 Participants

**FIGURE 1:**
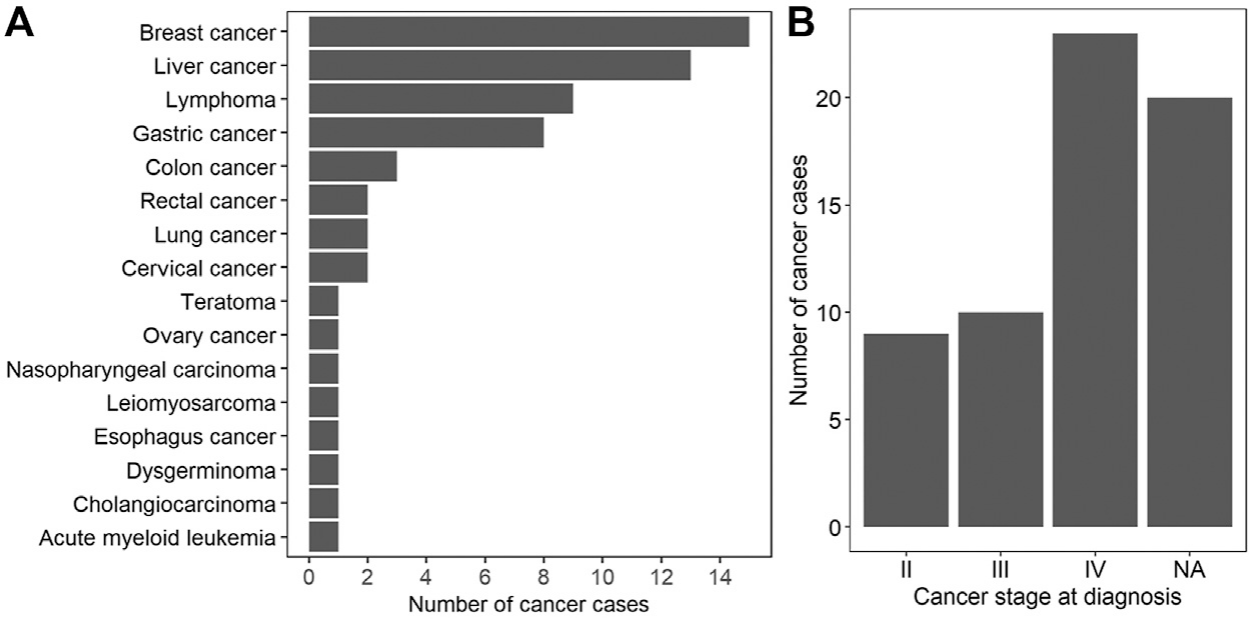
Characterization of 62 maternal cancer cases. **(A)** The number of cancer cases for each cancer type. **(B)** The distribution of cancer stages at diagnosis. NA: not available.

A total of 496 participants with MCA results between January 2016 and June 2019 at BGI labs were enrolled. The average age and gestational weeks of 496 participants were 31.8 years (SD, 5.51 years) and 17.1 weeks (SD, 3.38 weeks), respectively. All patients were interviewed, and the median follow-up time was 437 days (interquartile range 333–516). While the majority of interviewed participants remained asymptomatic, a total of 62 maternal cancer cases was clinically confirmed (Supplementary Tables S1, S2). At the time of NIPT, the mean age of 62 cancer patients was 33.48 years (SD, 5.72 years), and the mean gestational week was 17.95 (SD, 3.68 weeks). A wide spectrum of cancer types was detected from the 62 cases, with breast cancer (15 cases), liver cancer (13 cases), and lymphoma (9 cases), the most common cancer types (Figure 1A and Supplementary Tables S1, S2). With regard to cancer staging, 9, 10, and 23 patients were diagnosed at stages II, III, and IV respectively, whereas the cancer staging of the other 20 cases was unknown (Figure 1B and Supplementary Tables S1, S2). Among the 57 cancer cases of whom the time of final clinical diagnosis was available, the time from NIPT to the diagnosis of cancer ranged from 0 to 366 days, with a median duration of 78 days (interquartile range 35–167 days, Supplementary Tables S1, S2).

### The Patterns of Chromosomal Abnormalities Varied Greatly Between Cancer Types

**FIGURE 2:**
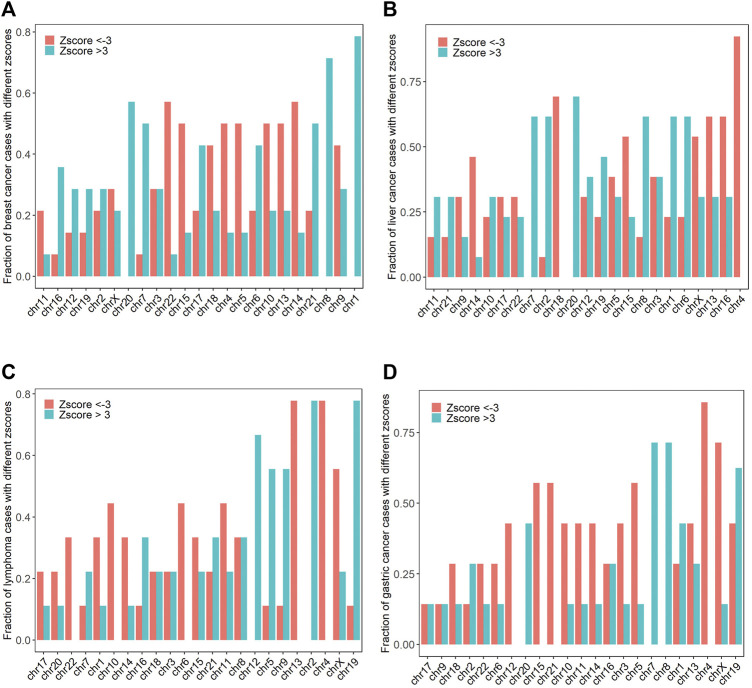
The frequencies of chromosomal amplifications (z score >3) and deletions (z score < −3) in breast cancer, liver cancer, lymphoma, and gastric cancer **(A–D)**.

Chromosome amplification and deletion are the most common structural chromosome abnormalities, which occur in 88% of cancer samples. In order to investigate the chromosomal abnormalities of the 62 maternal cancer cases, we computed the fraction of cancer cases with absolute z scores >3 for breast cancer, liver cancer, lymphoma, gastric cancer, and other cancer types. Chr1, chr8, chr20, chr7, and chr21 were the top five most frequently amplified chromosomes, while chr14, chr22, chr4, chr5, and chr10 were the top five most frequently deleted chromosomes in breast cancer (chromosome abnormality frequencies >50% for all cases). Chr20, chr1, chr2, chr6, and chr7 were the top five most frequently amplified chromosomes, with frequencies of 69.2, 61.5, 61.5, 61.5, and 61.5%, respectively. While chr4, chr18, chr13, chr16, and chr15 were the top five most common deletions in liver cancer, with frequencies of 92.3, 69.2, 61.5, 61.5, and 53.8%, respectively. The frequent chromosomal amplifications occurred at chr2, chr19, chr12, chr5, and chr9, while the common chromosomal deletions occurred at chr4, chr13, chrX, chr6, and chr10 in lymphoma (chromosome abnormality frequencies >44.4% for all cases). Commonly amplified chromosomes were chr7, chr8, chr19, chr1, and chr20, while commonly deleted chromosomes were chr4, chrX, chr5, chr15, and chr21 in gastric cancer (chromosome abnormality frequencies >42.9% for all cases, Figure 2).

### The Plasma Tumor Marker Test Shows Poor Performance in the Identification of Maternal Cancers

We performed the PTM test on 56 maternal cancer cases and 451 non-cancer participants and investigated whether PTMs might be used for identifying maternal cancer patients. First, we compared the PTM expression levels between cancer and normal participants and found that the PTM expression levels were significantly increased in maternal cancer as compared to non-cancer participants except for CA72-4 (*p* < 0.05 for all cases, Wilcoxon sum rank test, Supplementary Figure S2). Then, we aimed to analyze whether PTMs alone could effectively predict maternal cancer. Participants were considered at high risk for maternal cancer when the concentration of at least one PTM exceeded the prespecified cutoff value. The PTM test showed a sensitivity of 66.07% (95% confidence interval [CI], 52.19–78.19%) and specificity of 93.13% (95% CI, 90.39–95.28%) in identifying maternal cancers, suggesting the PTM test itself is not a good screening method (Supplementary Table S3).

### MTOP5Zscores are Established as a Feasible Method in Detection of Maternal Cancer

**FIGURE 3:**
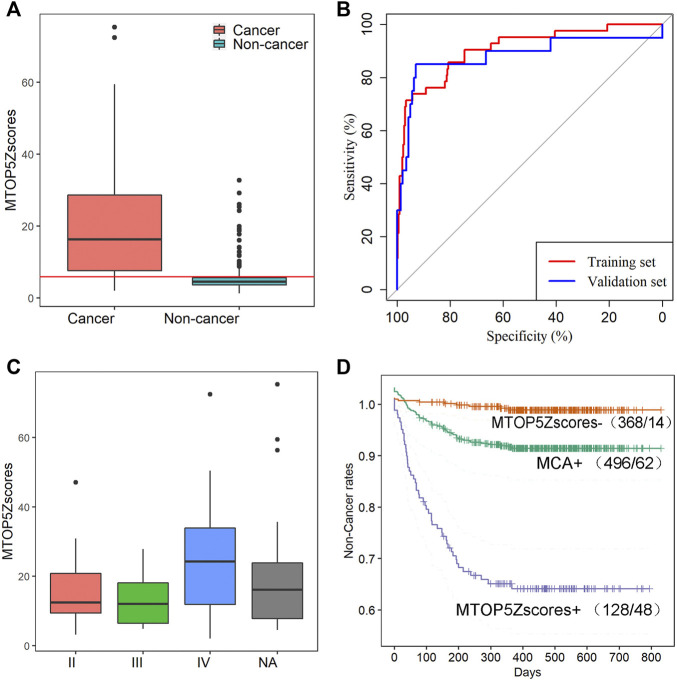
MTOP5Zscore analyses of 62 cancer cases in this study. **(A)** The comparison of MTOP5Zscores between 62 maternal cancer cases and 434 non-cancer participants; the red line represents the cutoff of 5.94 to determine MTOP5Zscore-positive. **(B)** The ROCs for MTOP5Zscores in the training and validation sets. **(C)** The comparison of MTOP5Zscores in maternal cancer patients at different cancer stages. **(D)** The Kaplan–Meier plot shows non-cancer rates for MCA-positive, MTOP5Zscore-positive, and MTOP5Zscore-negative groups.

A total of 496 participants with MCA results via NIPT were successfully interviewed in this study, and 62 maternal cancers were confirmed, giving a positive predictive value (PPV) of 12.5% for MCA. It demonstrates that MCA results alone showed unsatisfactory performance for cancer identification; therefore, we developed a new method named the mean of the top five chromosome z scores (MTOP5Zscores) to identify maternal cancer cases in pregnant women with MCA results. To determine whether MTOP5Zscores of maternal cancer patients deviate from non-cancer participants, we compared the differences of MTOP5Zscores between 62 cancer patients and 434 non-cancer participants who showed positive MCA results during NIPT, but no cancer was identified after follow-ups. The mean maternal age was 33.48 (SD, 5.72) years in 62 cancer cases and 31.6(SD, 5.45) years in the non-cancer group. The mean gestational age was 17.95 (SD, 3.68) weeks in the cancer group and 17.01(SD, 3.33) weeks in the non-cancer group. The age and gestational weeks of the cancer group were statistically higher than the non-cancer group (*p* < 0.05 for all cases, Wilcoxon rank sum test, Supplementary Table S4). As shown in Figure 3A, cancer patients showed significantly higher MTOP5Zscores than non-cancer participants (*p* < 0.0001, Wilcoxon sum rank test, Figure 3A). These results suggest that genomic stability in maternal cancer patients was severely disrupted.

To develop the MTOP5Zscore model, we assigned 42 cancer patients and 294 non-cancer participants enrolled between January 2016 and December 2017 to the training set, while 20 cancer patients and 140 non-cancer participants enrolled between January 2018 and June 2019 to the validation set. Next, we built ROC curves for MTOP5Zscores in the training and validation sets. The AUCs were 90.56 and 88.14% for MTOP5Zscores, respectively (Figure 3B). The optimal cutoff value of MTOP5Zscores was selected as 5.94 with a sensitivity of 85.71% (95% CI, 71.46–94.57%) and a specificity of 80.27% (95% CI 75.26–84.67%) (Table 1). Therefore, a patient was considered as MTOP5Zscore-positive if the patient had MTOP5Zscores >5.94 in the NIPT. Overall, 94 participants were reported as MTOP5Zscore-positive, 36 of whom were diagnosed with maternal cancer until the last day of the follow-up. Six cancer patients were MTOP5Zscore-negative but confirmed with maternal cancer (Table 1). In order to further assess the performance of MTOP5Zscores in the identification of maternal cancer, we used 20 cancer patients and 140 non-cancer participants in the validation set for validation analysis. MTOP5Zscores identified 17 out of 20 maternal cancer cases, giving an overall sensitivity of 85% (95% CI 62.11%–96.79%). Twenty-eight false-positive calls were confirmed after the follow-up (specificity 80%) (95% CI 72.41%–86.28%) (Table 1).

**TABLE 1:** The performances of MTOP5Zscores in the identification of maternal cancer in the training and validation sets.

	Training set		Validation set	
	*Cancer*	Non-cancer	*Cancer*	Non-cancer
Predicted cancer	36	58	17	28
Predicted non-cancer	6	236	3	112
Sensitivity	85.71% (71.46–94.57%)		85% (62.11–96.79%)	
Specificity	80.27% (75.26–84.67%)		80% (72.41–88.28%)	
PPV	38.3% (32.33–44.64%)		37.78% (29.36–47%)	
NPV	97.52% (94.93–98.81%)		97.39% (92.91–99.07%)	

Note, PPV, positive predictive value; NPV, negative predictive value. Numbers in the parentheses are 95% confidence intervals.

Lastly, the MTOP5Zscores were compared among the cancer patients with different stages, and no significant difference of MTOP5Zscores was observed across cancer stages (chi-squared = 1.3, *p*-value = 0.52, Kruskal–Wallis rank sum test, Figure 3C). Based on the follow-up data from January 2016 to June 2019, the MTOP5Zscore-positive group showed the lowest non-cancer rates in comparison with the MTOP5Zscores-negative group and MCA-positive group (*p*-value <0.05 for all cases, log-rank test, Figure 3D). The results suggest that participants with MTOP5Zscore-positive results have the highest risk of developing cancer, and a medical workup is highly recommended.

### Tumor Tissue Origin Classification

**FIGURE 4:**
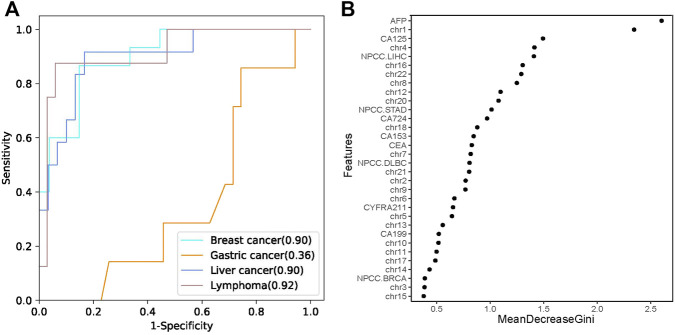
The tumor tissue origin classifier. **(A)** The performances of the random forest classifier estimated by leave-one-out cross validation. **(B)** The importance of different features in the random forest classifier. MeanDecreaseGini is the variable’s total decrease in node impurity measured by the Gini impurity criterion.

According to the statistics on cancer types in our study, the most frequent maternal cancer types are breast cancer, liver cancer, lymphoma, and gastric cancer. Therefore, we established a random forest model to classify the common cancer types using the NPCC values, z scores of 22 chromosomes, and seven PTMs as predictor variables (Supplementary Tables S5, S6). The sensitivity of the classifier was 93.33, 66.67, and 50%, while specificity was 66.67, 90, and 97.06% for the prediction of breast cancer, liver cancer, and lymphoma, respectively. The classifier predicted breast cancer, liver cancer, and lymphoma with positive predictive values (PPV) of 60.87, 72.73, and 80%, respectively (Table 2). The leave-one-out cross-validation result showed that the AUC values were 0.9, 0.9, and 0.92 for breast cancer, liver cancer, and lymphoma, respectively (Figure 4A), while the classifier performed poorly in predicting gastric cancer (AUC 0.36, Supplementary Figure S4). Moreover, we analyzed the feature importance in the random forest classifier and found that AFP, z scores of chr1, CA-125, z scores of chr4, and the NPCC value of LIHC were the top five most important features in the classifier (Figure 4B). These results indicate that the random forest classifier could effectively predict the tumor origin of maternal breast cancer, liver cancer, and lymphoma.

**TABLE 2:** The performance of the tumor tissue origin classifier for breast cancer, gastric cancer, liver cancer, and lymphoma.

	Breast cancer	Gastric cancer	Liver cancer	Lymphoma
Predicted breast cancer	14	5	2	2
Predicted Gastric	0	0	2	1
Predicted liver cancer	1	1	8	1
Predicted lymphoma	0	1	0	4
Sensitivity (95% CI)	93.33% (66.03–99.65%)	0% (0–43.91%)	66.67% (35.44–88.72%)	50% (17.45–82.55%)
Specificity (95% CI)	66.67% (46.02–82.76%)	91.43% (75.81–97.76%)	90% (72.32–97.38%)	97.06% (82.95–99.85%)
PPV (95% CI)	60.87% (38.78–79.53%)	0% (0–69%)	72.73% (39.32-%-92.67%)	80% (29.88–98.95%)
NPV (95% CI)	94.74% (71.89–99.72%)	82.05% (65.89–91.9%)	87.1% (69.24–95.78%)	89.19% (73.64–96.48%)

Note: CI, confidence intervals.

## Discussion

Over the past decade, the number of NIPT has been exploding throughout the world. With the rapid expansion of NIPT, failed and unexpected abnormal NIPTs are also ubiquitously observed in line with previous studies (Osborne et al., 2013; Bianchi et al., 2015a; Amant et al., 2015; Janssens et al., 2016; Dharajiya et al., 2018). A total of 62 cases of various maternal cancers were found in 496 participants with MCA identified via NIPTs in this study, suggesting that MCA is associated with maternal cancer via NIPT. Given MCA has a PPV of 12.5% (62/496), MCA alone is still unsatisfactory to be an indicator of cancer risk due to high false-positive results. Therefore, new methods are imminently needed to boost the effectiveness of maternal cancer identification besides MCA.

So far, more than 110 cases of maternal cancer have been found in failed or abnormal NIPTs. One major concern regarding incidental cancer cases identified by NIPT is whether NIPT could identify maternal cancer at earlier stages. Nine maternal cancer cases were diagnosed at stage II in this study, and 3 cases of maternal cancer at stage II were reported in Bianchi’s study (Bianchi et al., 2015a), suggesting NIPT may be competent to identify early-stage cancer patients. We previously developed a bioinformatics algorithm called the cancer detection pipeline (CDP) to identify maternal malignancies using genome profiling of copy-number variations. The performance can be further improved by incorporating CDP with plasma tumor markers (Ji et al., 2019). However, the pipeline involves complex bioinformatics analytical procedures, including mapping of raw sequencing data, CNV detection by using HMMcopy software, and calculation of the FCNV (Fraction of significant copy-number variation) score, which restrains its utility in clinical settings. This study established a method named MTOP5Zscores to identify maternal malignancies among pregnant women with MCA results. MTOP5Zscores calculated the mean of the top five chromosomes with the largest absolute z scores except for chromosome Y and 19 instead of selected chromosomal gains or losses (Cohen et al., 2016) or more aneuploidies involving chromosomes 13, 18, 21, X, or Y (Bianchi et al., 2015a) as seen in other studies. So MTOP5Zscores can capture the change in the genomic landscape in a more comprehensive manner. As compared to the CDP method, MTOP5Zscores directly uses z scores from NIPTs and effectively identifies incidental maternal malignancies among pregnant women who had MCA in NIPTs. No additional costs of sequencing or experiments were involved in the analysis. The method is simple and easily appliable for NIPT service providers and health-care professionals. Therefore, MTOP5Zscores further expanded the use of NIPT in the detection of occult maternal cancers during pregnancy beyond screening for fetal trisomy-21,-13, and -18 without extra cost.

Despite the encouraging utility of MTOP5Zscores, it has an obvious drawback that MTOP5Zscores could not tell the primary tumor based on abnormal z scores. To address this problem, we established a random forest model to classify the three common cancer types using the NPCC values, z scores of 22 chromosomes, and seven PTMs as predictor variables. The leave-one-out cross-validation result showed the classifier is robust and accurate for classifying breast cancer, liver cancer, and lymphoma. Moreover, AFP, z scores of chr1, CA-125, z scores of chr4, and the NPCC value of LIHC were the top five most important features in the classifier. AFP is a well-established tumor marker for liver cancer (Zhao et al., 2013). Elevated CA-125 and CEA are frequently observed in breast cancer samples (Fang et al., 2017; Gaughran et al., 2020). Previous studies have reported that breast cancer, liver cancer, and lymphoma samples showed distinct copy-number variation profiles (Cancer and Atlas, 2012; Ally et al., 2017). Our study also validated that chromosomal abnormalities varied considerably across cancer types. For instance, deletion of chr16 frequently occurs in liver cancer, and chr22 deletion is common in breast cancer. Therefore, these features are critical to the predictive capability of the random forest classifier.

In our study, the MTOP5Zscores method showed high sensitivity and specificity in the identification of maternal malignancies, which outperformed PTMs and our previous CDP model. The random forest classifier could predict the tumor origin of maternal breast cancer, liver cancer, and lymphoma with high accuracy. Although this study paves a way for pre-symptomatic detection of maternal cancer and provides evidence-based recommendations for the obstetricians to make optimal decisions when MCAs were reported, this study still has a few limitations. First, MTOP5Zscores effectively identified cancer patients among pregnant women with MCA results found with NIPTs. However, MTOP5Zscores is not a method to screen for maternal cancer in all pregnant women who undergo NIPTs. Second, the number of samples is relatively small in the validation dataset, and further validation of the accuracy of MTOP5Zscores in a larger size of cancer samples is needed in further studies. Third, the random forest classifier still couldn’t specify the exact origin of circulating tumor DNAs for most cancer types in the study; therefore, other diagnostic approaches such as whole-body magnetic resonance imaging may be needed for MTOP5Zscore-positive pregnant women in clinical settings (Amant et al., 2015; Peccatori et al., 2017). The MTOP5Zscore method may need to be used in combination with DNA methylation signatures to better identify the primary tumor. One of the major ethical issues with suspected maternal cancer identified by MCA results by NIPT is how to accurately interpret the abnormal results and cautiously transmit the information of cancer risk to the providers and the patients. In this study, MTOP5Zscore-positive participants had a PPV of 37.78% and the lowest non-cancer rate, suggesting a medical workup may be needed for this category of pregnant women, and they should be well informed of their cancer risks. MTOP5Zscore-negative participants had a negative predictive value (NPV) as high as 95.89%. Therefore, a postnatal NIPT is suggested to reassure the care providers and the patients.

## Conclusion

In summary, the MTOP5Zscore method shows strong clinical utility to detect pre-symptomatic maternal cancer using z scores generated via NIPT. In addition, the random forest classifier could effectively predict the tumor origin of maternal breast cancer, liver cancer, and lymphoma. The study reported here lays the foundation for future application of NIPT to identify maternal cancer in addition to screening for fetal aneuploidies in clinical practice.

## Data Availability

The datasets presented in this study can be found in online repositories. The names of the repository/repositories and accession number(s) can be found in the article/Supplementary Material.
