# Supplementary material for: Effective Identification of Maternal Malignancies in Pregnancies Undergoing Noninvasive Prenatal Testing
Source: Front Genet. 2022 Feb 10;13:802865. doi: 10.3389/fgene.2022.802865 (PMC8900746; doi:10.3389/fgene.2022.802865)
Supplement: Supplementary file 1 [file DataSheet1.docx]

**Supplementary Method1. The questionnaire to investigate the patients’ health status after NIPTs**

Please read the survey carefully and give objective answers.

| Company of NIFTY test: |  | Patient’s name: |  |
| --- | --- | --- | --- |
| Hospital of childbirth: |  | Patient’ age: |  |

REVIEW OF CHILD’S HEALTH

1.Did you take amniocentesis test after NIFTY tests? Yes No

If "yes", please describe the results of amniocentesis test:

2.Were you able to give birth?

Yes Child’s birth date______________ No

3.Does your baby have regular physical examinations? Yes No

4.Is your baby developing normally? Yes No

REVIEW OF PATIENT’S HEALTH

(If you answer “Yes” to any of these questions, please provide further details in the space below each question:

General:

1. How would you assess your overall health picture in the past year?

__________________________________________________________________________________________________________________________________________________________________________________________________________________________________________________________________________________________________________________

2. Have you been to the hospital in the past year? because of other diseases or physical problems? __________________________________________________________________________________________________________________________________________________________________________________________________________________________________________________________________________________________________________________

Head:

1. Have you suffered from headaches in the past year? Yes No

If so, have they been "labeled" (i.e. migraines, tension, cluster, etc.)

__________________________________________________________________________________________________________________________________________________________________________________________________________________________________________________________________________________________________________________

2. Have you had ringing in your ears, hearing loss, nasal obstruction, nose bleeding, occasional dizziness in the past year? Yes No

If "yes", please describe:

__________________________________________________________________________________________________________________________________________________________________________________________________________________________________________________________________________________________________________________3. Is there a past history of acoustic trauma, ear disease, or family history of a hearing deficit? Yes No

If "yes", please specify:

__________________________________________________________________________________________________________________________________________________________________________________________________________________________________________________________________________________________________________________

4. Have there been any changes in your vision in the past year? Yes No

If so, in which eye and for how long?

__________________________________________________________________________________________________________________________________________________________________________________________________________________________________________________________________________________________________________________5. Have you had hoarseness, other recurrent abnormalities of voice in the past year? Yes No

6.Have you had continuous dry cough, sputum with blood, repeated pneumonia in the past year? Yes No

If "yes", please specif: __________________________________________________________________________________________________________________________________________________________________________________________________________________________________________________________________________________________________________________

Neck:

1. Have you had neck pain or stiffness in the past year? Yes No

If so, are there provoking factors? __________________________________________________________________________________________________________________________________________________________________________________________________________________________________________________________________________________________________________________

2. Have you had swollen glands in the neck in the past year? Yes No

If so, are they associated with a sore throat, or other signs of infection? __________________________________________________________________________________________________________________________________________________________________________________________________________________________________________________________________________________________________________________

3. Have you had thyroid enlargement (goiter), or neck tenderness in the past year?

Yes No

If "yes", please specify: __________________________________________________________________________________________________________________________________________________________________________________________________________________________________________________________________________________________________________________

Lymphatic System:

1. Have you had persistent swollen glands of the neck, underarms, groin or thighs in the past year? Yes No

If yes, please describe: __________________________________________________________________________________________________________________________________________________________________________________________________________________________________________________________________________________________________________________

Chest:

1. Have you had chest pain, shortness of breath, asthma, emphysema, COPD, cough, chest congestion wheezing, or diminished exercise tolerance in the past year?

Yes No

If yes, please describe: __________________________________________________________________________________________________________________________________________________________________________________________________________________________________________________________________________________________________________________

2.Have you felt any discomfort swallowing, pains or abnormal feelings in

your esophagus, or choking sensations in the past year? Yes No

If yes, please describe: __________________________________________________________________________________________________________________________________________________________________________________________________________________________________________________________________________________________________________________

3.Have you had abnormal breast tumor, nipple secretions, lymph node enlargement in the past year? Yes No

If yes, please describe: __________________________________________________________________________________________________________________________________________________________________________________________________________________________________________________________________________________________________________________

Abdomen:

1. Have you had chronic or recurrent abdominal pain, indigestion, nausea, vomiting, diarrhea, constipation or previous endoscopy procedures in the past year?

Yes No

2. Have you had belching of stomach acid, severe or recurrent "heartburn" in the past year? Yes No

If so, please list provoking factors: __________________________________________________________________________________________________________________________________________________________________________________________________________________________________________________________________________________________________________________

3. Have you noted any change in bowel habits, such as dark stools, diminished caliber of the stool, straining at defecation, or a persistent feeling of the need to evacuate the bowel unrelieved by passage of stool in the past year? Yes No

If yes, please describe: __________________________________________________________________________________________________________________________________________________________________________________________________________________________________________________________________________________________________________________

4. Have you had prior colonoscopy, flexible sigmoidoscopy or upper endoscopy (EGD) in the past year? Yes No

If yes, when did you have it and what did it show? __________________________________________________________________________________________________________________________________________________________________________________________________________________________________________________________________________________________________________________

Genitourinary Tract:

1. Have you had diminished size and force of the urinary stream in the past year? Yes No

If yes, please describe:

__________________________________________________________________________________________________________________________________________________________________________________________________________________________________________________________________________________________________________________2.Have you had non-menstrual vaginal bleeding, pink vaginal secretions, bleeding after sexual intercourse, bloody vaginal discharge, in the past year? Yes No

If yes, when did you have it and what did it show? __________________________________________________________________________________________________________________________________________________________________________________________________________________________________________________________________________________________________________________

3.Have you had frequent urination, dysuria, painless, blood in your urine, back pain (dull pain) in the past year? Yes No

If yes, please describe: __________________________________________________________________________________________________________________________________________________________________________________________________________________________________________________________________________________________________________________

Skin:

1. Have you noticed moles, rapidly growing warts, skin discoloration, hair loss, itching, skin ulcers or other symptoms in the past year?

Yes No

If yes, when did you have it and what did it show? __________________________________________________________________________________________________________________________________________________________________________________________________________________________________________________________________________________________________________________

Extremities:

1. Have you had chronic or recurrent joint pain, swelling, stiffness or redness in the past year. Yes No

2. Have you had unexplained weight loss in the past year? Yes No

If yes, please specify the exact loss of weight (Kg)____________________________

3.Have you noticed any growths on your skin, breasts, tongue, or any other body part in the past year? Yes No

If yes, please specify____________________________________________________

Central Nervous System:

1. Have you had motor or sensory abnormalities of any area of the body in the past year? Yes No

If yes, please specify____________________________________________________

2. Have you had unusual levels of anxiety or depression in the past year?

Yes No

If yes, please specify____________________________________________________

Family history

1. Have you or anyone in your immediate family (parents, grandparents, children, siblings) ever had any of the following conditions?

Cancer Yes No

Genetic diseases Yes No

Blood diseases Yes No

Other major diseases Yes No If yes, please specify____________________

Other Pertinent Medical Information:

1. Are there other points that you feel should be included in your medical history?

__________________________________________________________________________________________________________________________________________________________________________________________________________________________________________________________________________________________________________________

**Supplementary Method2. The questionnaire to collect the medical information of the patients with maternal cancer**

1. In the past year, have you been diagnosed with malignant cancer?

Yes Cancer name： No

2. What’s the exact diagnosis date of your cancer：_____________

3. What are the initial symptoms of your cancer：_____________

4. What’s the clinical stage of your cancer：_____________

5. Does your cancer metastasize to surrounding lympho nodes or distant organs?

Yes Metastatic sites: No

6. Have you received surgical treatment?

Yes Surgery date and name: No

7. Have you received chemotherapy?

Yes The current course of chemotherapy:__________ the total number of courses of chemotherapy: No

8. Have you received radiotherapy?

Yes The current course of radiotherapy:__________ the total number of courses of radiotherapy: No

9. Do you agree to receive NIFTY test to evaluate the risk of recurrence or distant metastasis?

Yes No

10. Do you have family cancer history?

Yes Cancer name： No

**Supplementary Table1. Demographic factors, cancer risk evaluation and clinical characteristics for the 41 maternal cancer cases in the training set**

| **Case ID** | **Age** | **Gestational weeks** | **Cancer risk evaluation by MTOP5Zscores** | **MTOP5Zscores** | **Cancer type** | **Cancer stage** | **Distant metastasis** | **Treatment** | **Clinical outcome** | **Time from NIPT to clinical diagnosis of cancer(Days)** |
| --- | --- | --- | --- | --- | --- | --- | --- | --- | --- | --- |
| MCR2809C | 34 | 17 | MTOP5Zscores+ | 25.08 | Breast cancer | IV | Bone | Chemotherapy | Alive | 65 |
| MCR2598C | 36 | 17 | MTOP5Zscores- | 3.14 | Breast cancer | II | None | Chinese medicine | Alive | 0^a^ |
| MCR1266C | 36 | 15 | MTOP5Zscores+ | 12.44 | Breast cancer | IIb | None | Chemotherapy | Alive | 51 |
| MCR0336C | 46 | 28 | MTOP5Zscores+ | 6.02 | Breast cancer | IIb | None | Chemotherapy | Alive | 149 |
| MCR1189C | 40 | 15 | MTOP5Zscores+ | 17 | Breast cancer | IIIa | Left axillary lymph node and cervical lymph nodes | Alive | Alive | 36 |
| MCR1260C | 37 | 17 | MTOP5Zscores+ | 18.31 | Breast cancer | IV | Brain, liver,  lung | Conservative  treatment | Alive but unconscious | 95 |
| MCR1804C | 35 | 17 | MTOP5Zscores+ | 11.24 | Left breast cancer | Ⅲb | Right breast | Surgery,  Chemotherapy | Alive | 60 |
| MCR1105C | 32 | 17 | MTOP5Zscores+ | 15.15 | Relapse of breast cancer | IV | Bone | Surgery,  chemotherapy | Alive | 32 ^a^ |
| MCR2693C | 42 | 14 | MTOP5Zscores+ | 31.28 | Relapse of breast cancer | IV | Spine, ribs and hip | Surgery, Chemotherapy, Radiotherapy, Hormonal therapy | Alive | 40 ^a^ |
| MCR1124C | 34 | 21 | MTOP5Zscores+ | 18.45 | Breast cancer | III | Axillary lymph node | Surgery,  Chemotherapy | Alive | 17 |
| MCR0284C | 36 | 13 | MTOP5Zscores+ | 47.1 | Liver cancer | II | None | Surgery,  Chemotherapy | Alive | 90 |
| MCR1448C | 30 | 33 | MTOP5Zscores+ | 35.78 | Liver cancer | IV | Bone | Conservative treatment | Alive | 0^a^ |
| MCR1661C | 23 | 23 | MTOP5Zscores- | 4.81 | Liver cancer | III | None | Surgery | Alive | 157 |
| MCR1669C | 36 | 16 | MTOP5Zscores+ | 27.82 | Liver cancer | IIIc | None | Transarterial Chemoembolization(TACE) | Alive | 194 |
| MCR1361C | 33 | 22 | MTOP5Zscores+ | 49.92 | Liver cancer | IV | Portal vein | Conservative  treatment | Dead | 41 |
| MCR1254C | 24 | 18 | MTOP5Zscores+ | 15.59 | Liver cancer | II | None | Surgery | Alive | 21 |
| MCR1327C | 40 | 21 | MTOP5Zscores- | 5.67 | Liver cancer | NA | NA | Chemotherapy | Alive | NA |
| MCR1049C | 42 | 17 | MTOP5Zscores+ | 6.12 | Liver cancer | IIIb | None | Surgery, TACE | Alive | 192 |
| MCR1376C | 23 | 20 | MTOP5Zscores+ | 5.97 | Liver cancer | IV | Lung | Surgery, TACE chemotherapy | Alive | 214 |
| MCR2689C | 33 | 13 | MTOP5Zscores+ | 30.82 | Lymphoma | II | None | Chemotherapy | Alive | 115 |
| MCR0152C | 22 | 15 | MTOP5Zscores- | 13 | Lymphoma | NA | NA | NA | Alive | NA |
| MCR0364C | 26 | 20 | MTOP5Zscores+ | 32.1 | [Lymphoma](https://www.baidu.com/link?url=KDO3l4Y7McgFajCaEJhdXVA8UXoEmR73mbEqyP6d4cEvjfz_WHb_o1Cjz0n8WYbciu3lXny2pXtP8xi8Mtycw81RJuzR7S57jgjml0ne5RjUOYTb-4lntnIFNfz7tSyz3R3r9LnvPTZNIyhJiv_Rpq&wd=&eqid=a20fb8730001529800000006597a9e78) | IV | Liver, spleen, abdominal cavity, cervical lymph nodes | Chemotherapy | Alive | 366 |
| MCR0353C | 25 | 20 | MTOP5Zscores+ | 23.2 | Lymphoma | NA | None | Chemotherapy | Alive | 182 |
| MCR1177C | 31 | 18+ | MTOP5Zscores+ | 6.06 | Lymphoma | IV | Lung | Surgery,  Chemotherapy | Dead | 203 |
| MCR1661C | 27 | 19 | MTOP5Zscores- | 4.05 | Lymphoma | IVa | Lung | Chemotherapy | Alive | 355 |
| MCR2106C | 32 | 17 | MTOP5Zscores+ | 12.34 | Lymphoma | II | None | Chemotherapy | Alive | 163 |
| MCR1532C | 40 | 16 | MTOP5Zscores+ | 20.53 | Lymphoma | NA | NA | NA | Alive | 178 |
| MCR4966C | 32 | 23 | MTOP5Zscores+ | 18.71 | Lymphoma | III | Thoracic cavity abdominal cavity | Chemotherapy | Alive | 167 |
| MCR0178C | 32 | 13 | MTOP5Zscores+ | 32.02 | Colon cancer | IV | Liver and lung | Chemotherapy | Alive | 11 |
| MCR5022C | 38 | 12 | MTOP5Zscores+ | 28.92 | Colon cancer | IV | Liver | Surgery,  Chemotherapy | Alive | 238 |
| MCR1219C | 36 | 20 | MTOP5Zscores+ | 24.26 | Gastric cancer | IV | Lung, abdominal cavity | Conservative treatment | Dead | 9 |
| MCR1969C | 40 | 20 | MTOP5Zscores- | 5.66 | Gastric cancer | III | Gastric lymph nodes | Surgery,  Chemotherapy | Alive | 330 |
| MCR1460C | 29 | 17 | MTOP5Zscores+ | 25.71 | Gastric cancer | IV | Liver | Conservative  treatment | Alive | 115 |
| MCR4661C | 44 | 15 | MTOP5Zscores- | 4.99 | Gastric cancer | NA | NA | Conservative  treatment | Alive | 12 |
| MCR0989C | 42 | 18 | MTOP5Zscores+ | 50.41 | Rectal cancer | IV | Liver and lung | Chemotherapy | Alive | 99 |
| MCR1443C | 37 | 28+ | MTOP5Zscores+ | 72.44 | Cervical cancer | IV | Right internal iliac lymph node and ovary | Surgery | Dead | 0^a^ |
| MCR1170C | 28 | 13 | MTOP5Zscores+ | 20.8 | Dysgerminoma of ovary | II | None | Surgery | Alive | 22 |
| MCR0419C | 29 | 26 | MTOP5Zscores+ | 14.22 | Relapse of teratoma of ovary | IV | Inguinal lymph nodes | Surgery,  Chemotherapy | Alive | 200 ^a^ |
| MCR1700C | 24 | 18 | MTOP5Zscores+ | 7.27 | Leiomyosarcoma of mandible | III | None | Surgery,  Chemotherapy | Alive | 117 |
| MCR4713C | 37 | 19 | MTOP5Zscores+ | 17.11 | Lung cancer | IV | Lymph nodes and bones | Chemotherapy | Alive | 77 |
| MCR1489C | 34 | 18 | MTOP5Zscores+ | 8.5 | [Nasopharyngeal carcinoma](https://www.baidu.com/link?url=SI_59qgzS7CDzxe4cxgbjmzD5LkpbOcOWk3qxifPNz1gILNJkHTT2oLEGYs8-8RAMlVWwGqe9etFwIkt5JgLIuWFodux-6n5WlIVmneN4YCxLWbmsXBmGHw-fv01gE4I&wd=&eqid=f4939766000374e2000000065be18d0b) | NA | NA | Chemotherapy, radiotherapy | Alive | 270 |
| MCR1418C | 29 | 20 | MTOP5Zscores+ | 23.1 | Acute myeloid leukemia | NA | NA | Chemotherapy  transplant | Alive | NA |

^a^ the patients were diagnosed with maternal cancer prior to NIPT tests.

**Supplementary Table2. Demographic factors, cancer risk evaluation and clinical characteristics for the 20 maternal cancer cases in the test set**

| **Case ID** | **Age** | **Gestational weeks** | **Cancer risk evaluation by MTOP5Zscores** | **MTOP5Zscores** | **Cancer type** | **Cancer stage** | | **Distant metastasis** | **Treatment** | **Clinical outcome** | **Time from NIPT to clinical diagnosis of cancer(Days)** |
| --- | --- | --- | --- | --- | --- | --- | --- | --- | --- | --- | --- |
| MCR3351C | 42 | 18 | MTOP5Zscores+ | 7.1 | Esophagus cancer | IV | NA | | Conservative treatment | Dead | 75 |
| MCR3565C | 42 | 16 | MTOP5Zscores+ | 7.86 | Rectal cancer | NA | NA | | NA | Alive | 38 |
| MCR4882C | 34 | 18 | MTOP5Zscores+ | 11.78 | Cholangiocarcinoma | NA | NA | | NA | Alive | NA |
| MCR4803C | 33 | 15 | MTOP5Zscores+ | 11.7 | Breast cancer | NA | NA | | None | Alive | 46 |
| MCR3356C | 34 | 18 | MTOP5Zscores+ | 9.53 | Lung cancer | IV | Lymph nodes | | Chemotherapy | Alive | 42 |
| MCR2387C | 32 | 17 | MTOP5Zscores+ | 75.31 | Liver cancer | NA | NA | | NA | Alive | 161 |
| MCR3449C | 29 | 18 | MTOP5Zscores+ | 9.36 | Ovary cancer | II | None | | Surgery+chemotherapy | Alive | 292 |
| MCR2402C | 38 | 16 | MTOP5Zscores+ | 12.74 | cervical cancer | III | None | | Surgery,chemotherapy,Radiotherapy | Alive | 106 |
| MCR3067C | 36 | 24 | MTOP5Zscores+ | 7.54 | Relapse of breast cancer | NA | NA | | Surgery,chemotherapy | Alive | 0 |
| MCR2694C | 33 | 16 | MTOP5Zscores+ | 44.71 | Gastric cancer | IV | NA | | Conservative treatment | Dead | 35 |
| MCR3313C | 34 | 18 | MTOP5Zscores+ | 20.12 | Liver cancer | NA | NA | | NA | Alive | 30 |
| MCR2352C | 33 | 16 | MTOP5Zscores- | 5.38 | Gastric cancer | NA | NA | | NA | Alive | 78 |
| MCR2348C | 38 | 17 | MTOP5Zscores+ | 35.7 | Breast cancer | IV | bone,liver | |  | Dead | 68 |
| MCR2387C | 33 | 15 | MTOP5Zscores+ | 14.52 | Gastric cancer | IV | NA | | Conservative treatment | Dead | 31 |
| MCR4035C | 40 | 17 | MTOP5Zscores- | 4.51 | Breast cancer | NA | NA | | NA | Alive | 193 |
| MCR2781C | 31 | 16 | MTOP5Zscores+ | 59.41 | Colon cancer | NA | NA | | NA | Alive | 0 |
| MCR6672C | 37 | 20 | MTOP5Zscores+ | 35.66 | Liver cancer | IV | NA | | Conservative treatment | Alive | 27 |
| MCR6966C | 40 | 18 | MTOP5Zscores+ | 56.31 | Liver cancer | IV | digestive tract | | Conservative treatment | Alive | 133 |
| MCR6800C | 33 | 16 | MTOP5Zscores+ | 19.23 | Breast cancer | NA | NA | | NA | Alive | NA |
| MCR3827C | 32 | 26 | MTOP5Zscores- | 2.06 | Gastric cancer | NA | NA | | NA | Alive | NA |

**Supplementary Table3. The performances of PTMs in the identification of maternal cancer in the whole dataset**

|  | Whole set | |
| --- | --- | --- |
|  | Cancer | Non-cancer |
| Predicted cancer | 37 | 31 |
| Predicted non-cancer | 19 | 420 |
| Sensitivity | 66.07% (95% CI, 52.19%-78.19%) | |
| Specificity | 93.13% (95% CI, 90.39%-95.28%) | |
| PPV | 54.41% (95% CI, 44.74%-63.76%) | |
| NPV | 95.67% (95% CI, 93.87%- 96.96%) | |

Note, PPV, positive predictive value, NPV, negative predictive value. Numbers in the parentheses are 95% confidence intervals (CI).

**Supplementary Table4. Statistical analyses of age, gestational weeks and MTOP5Zscores among 62 cancer patients, 434 non-cancer participants**

| **Group** |  |  |  |
| --- | --- | --- | --- |
|  | **Cancer**  **Mean (SD)** | **Non-cancer**  **Mean (SD)** | **P value (Wilcoxon sum rank test)** |
| Age | 33.48 (5. 72) | 31.6(5.45) | P < 0.05 |
| Gestational weeks | 17.95(3.68) | 17.01(3.33) | P < 0.05 |
| MTOP5Zscores | 21.03(17.08) | 5.18(3.44) | P < 0.0001 |

Cancer and non-cancer refer to 62 maternal cancer cases and 434 confirmed non-cancer participants.

**Supplementary Table5. The z scores of 22 chromosomes of maternal liver cancer, breast cancer and lymphoma cases in the random forest classifier**

| Sample | Cancer type | chr1 | chr2 | chr3 | chr4 | chr5 | chr6 | chr7 | chr8 | | chr9 | chr10 | chr11 | chr12 | chr13 | chr14 | chr15 | chr16 | chr17 | chr18 | chr20 | chr21 | chr22 | chrX |
| --- | --- | --- | --- | --- | --- | --- | --- | --- | --- | --- | --- | --- | --- | --- | --- | --- | --- | --- | --- | --- | --- | --- | --- | --- |
| MCR2809C | Breast cancer | 26.86 | -0.15 | 20.56 | 1.42 | -19.49 | -9.18 | -1.98 | 4.74 | -8.23 | | -18.89 | -6.83 | 6.37 | -23.17 | -6.25 | -17.61 | 2.55 | 7.35 | 21.30 | 11.18 | 6.03 | 3.45 | -33.50 |
| MCR2598C | Breast cancer | 4.48 | 1.10 | 0.48 | -0.06 | 0.46 | -1.31 | 0.16 | 0.23 | -1.90 | | 2.38 | -0.27 | -0.10 | -1.28 | 0.06 | 1.00 | -0.82 | 3.58 | -2.64 | 2.60 | -0.63 | -2.21 | -0.91 |
| MCR1266C | Breast cancer | 9.94 | 6.58 | 0.22 | -11.22 | 1.01 | 0.54 | 11.20 | 2.09 | 5.00 | | -1.76 | -0.30 | -0.14 | -12.40 | -17.45 | -6.86 | 0.59 | 8.40 | -1.12 | -2.49 | -1.70 | -0.77 | 4.75 |
| MCR0336C | Breast cancer | 5.60 | 1.75 | 2.51 | -6.30 | -6.40 | 5.30 | -1.32 | 6.49 | -0.22 | | 2.11 | 2.38 | -0.69 | 1.38 | -1.37 | -0.18 | -1.85 | -1.59 | -1.68 | 0.92 | -2.28 | -1.57 | 1.09 |
| MCR1189C | Breast cancer | 15.00 | 9.21 | -6.99 | 1.57 | -8.75 | 9.77 | 7.17 | 17.88 | -5.33 | | -6.87 | -8.20 | -12.44 | -18.26 | 12.18 | -12.17 | 7.91 | 19.07 | -8.40 | 9.82 | 8.07 | -14.78 | 9.13 |
| MCR1260C | Breast cancer | 1.74 | 4.35 | -12.05 | 4.61 | 5.43 | -11.54 | 27.16 | 30.07 | -5.94 | | -10.75 | -2.02 | 2.93 | 2.09 | -3.46 | 3.51 | -0.39 | 10.65 | -7.32 | 2.89 | 6.38 | -10.28 | 2.04 |
| MCR1804C | Breast cancer | 5.16 | 2.22 | -4.11 | -3.66 | 0.92 | 7.82 | -5.22 | 20.97 | 4.65 | | -0.67 | -0.23 | -2.31 | -6.74 | 1.42 | 0.24 | -5.13 | 12.98 | -7.69 | 0.33 | 6.11 | -4.96 | -2.92 |
| MCR1105C | Breast cancer | 7.36 | -5.40 | -3.11 | -1.94 | -5.84 | 16.36 | 14.58 | 18.48 | 4.35 | | -6.58 | -2.28 | -2.08 | -14.09 | -9.54 | -4.85 | 7.79 | -12.23 | -1.24 | 7.44 | 7.04 | -8.38 | 6.16 |
| MCR2693C | Breast cancer | 5.93 | -5.50 | 5.69 | 6.32 | -23.76 | -18.98 | 8.31 | 51.42 | -3.39 | | -6.94 | 4.23 | 4.46 | 11.09 | -18.79 | 7.46 | 10.06 | -26.84 | 4.54 | 4.74 | 23.32 | -20.04 | -31.05 |
| MCR1124C | Breast cancer | 9.12 | 1.11 | 9.92 | -20.29 | -23.37 | -0.38 | -2.35 | 18.11 | 8.03 | | 6.43 | 1.82 | 1.02 | 5.20 | -15.20 | -8.94 | 2.71 | -8.63 | 15.26 | 4.04 | -11.87 | -10.90 | 0.73 |
| MCR4803C | Breast cancer | 2.97 | -1.65 | -0.55 | -3.45 | 0.80 | -2.31 | 1.03 | 24.17 | -6.38 | | -4.55 | 2.63 | 3.20 | -12.04 | 4.11 | -0.60 | 7.07 | -2.14 | -1.51 | 5.42 | -3.22 | -8.84 | -0.29 |
| MCR3067C | Breast cancer | 4.46 | 5.22 | -2.92 | -1.14 | 4.69 | 2.04 | 6.11 | -0.36 | -0.08 | | -10.07 | -5.47 | 8.83 | -6.82 | -0.46 | -4.60 | -1.16 | -0.26 | -5.89 | 5.77 | 4.70 | -3.65 | -3.34 |
| MCR2348C | Breast cancer | 17.13 | -4.98 | -5.99 | -1.68 | -3.09 | -24.66 | -3.46 | 12.50 | -1.98 | | 4.44 | -34.39 | 17.15 | -31.32 | 22.19 | -6.38 | 19.13 | 8.76 | -10.09 | 17.95 | 16.42 | -5.06 | -65.93 |
| MCR4035C | Breast cancer | 2.09 | 0.00 | 1.74 | -5.60 | -4.40 | 4.13 | 0.70 | 2.67 | 0.85 | | 0.01 | -1.37 | -0.55 | 2.38 | -4.11 | -0.56 | 1.24 | -2.46 | -4.30 | 2.80 | 0.12 | -1.48 | -0.39 |
| MCR6800C | Breast cancer | 10.49 | -3.04 | 12.45 | -15.72 | -2.14 | 15.67 | 8.22 | 3.49 | -11.06 | | 4.04 | 0.78 | -3.44 | 9.77 | -10.60 | -22.72 | 19.89 | 2.72 | -22.13 | 4.10 | -7.94 | -1.37 | -8.51 |
| MCR0284C | Liver cancer | -24.56 | -16.11 | 3.58 | -59.01 | -2.97 | 13.15 | 14.83 | 25.31 | -8.60 | | -3.19 | 8.48 | 10.85 | 83.07 | -10.10 | 5.10 | 6.07 | -9.14 | -41.62 | 26.47 | -22.27 | 1.65 | 15.87 |
| MCR1448C | Liver cancer | 10.14 | 30.83 | 5.10 | -42.03 | -9.05 | 21.87 | 7.31 | 6.88 | -7.21 | | 5.05 | -14.75 | 8.67 | 18.06 | -42.29 | -12.85 | -41.88 | -6.07 | -1.12 | 12.84 | 3.43 | 16.11 | -11.11 |
| MCR1661C | Liver cancer | 3.44 | 5.47 | -2.91 | -4.46 | 0.67 | -3.03 | -0.46 | -0.69 | 0.20 | | 0.34 | 1.45 | 4.65 | -3.59 | -0.59 | -3.85 | -1.25 | -0.31 | 0.40 | 5.60 | -1.82 | 1.90 | 2.20 |
| MCR1669C | Liver cancer | 25.94 | 3.05 | -3.02 | -31.89 | 16.20 | 7.96 | 3.99 | -10.68 | -0.42 | | 1.99 | -7.79 | 11.28 | -29.22 | 0.97 | 3.55 | -35.37 | 3.94 | -16.67 | 11.33 | 5.05 | 3.36 | 6.54 |
| MCR1361C | Liver cancer | 13.97 | 17.38 | -3.87 | -37.35 | -10.55 | 18.00 | 11.72 | -6.54 | 13.26 | | 7.68 | 8.71 | 81.98 | -33.30 | -31.56 | -3.10 | -65.39 | 8.15 | -25.38 | 9.08 | 5.78 | 5.25 | 15.69 |
| MCR1254C | Liver cancer | 8.61 | -1.05 | 3.76 | -19.32 | 4.91 | 3.85 | 2.64 | 13.55 | 1.66 | | 13.46 | 0.67 | 1.73 | -8.97 | 1.40 | -14.61 | -17.01 | -3.10 | -12.00 | 2.88 | 1.96 | 1.92 | -8.18 |
| MCR1327C | Liver cancer | 4.48 | 0.43 | -0.90 | -4.19 | -0.95 | 3.30 | 1.17 | 8.27 | -0.85 | | -0.72 | 0.67 | -0.66 | -5.17 | 1.88 | -2.32 | -6.04 | -0.43 | 0.65 | 0.56 | -0.81 | -0.95 | 4.38 |
| MCR1049C | Liver cancer | 8.12 | 0.76 | 7.90 | -2.72 | 4.23 | 1.45 | 0.70 | -2.77 | 1.96 | | 0.97 | -2.21 | 0.64 | -1.93 | 1.66 | 0.01 | -5.37 | 2.64 | -4.95 | -0.84 | 1.26 | -0.89 | -4.25 |
| MCR1376C | Liver cancer | 2.68 | -1.97 | -0.42 | -6.89 | 1.07 | 2.49 | 1.91 | 8.52 | 0.64 | | 0.95 | 0.24 | 0.23 | -7.17 | -2.43 | -0.83 | -4.60 | -0.60 | 0.67 | -0.48 | 1.63 | 1.88 | -1.17 |
| MCR2387C | Liver cancer | -14.32 | 18.63 | -8.34 | -37.21 | -3.20 | 60.35 | 61.66 | 4.78 | 19.75 | | -54.56 | -2.65 | -10.31 | 125.81 | -44.57 | -31.86 | 11.63 | -0.25 | -48.53 | 74.17 | -0.52 | -28.72 | -7.57 |
| MCR3313C | Liver cancer | 1.62 | 15.07 | 23.87 | -8.02 | -21.01 | -23.78 | 14.69 | 16.87 | -1.50 | | -2.82 | 14.54 | -9.61 | -8.00 | 10.76 | -7.18 | 8.24 | -2.06 | -8.50 | 8.22 | -7.11 | -8.21 | -7.81 |
| MCR6672C | Liver cancer | 14.50 | 19.51 | -13.46 | -52.99 | 37.77 | -11.92 | 22.03 | -1.33 | -6.03 | | 26.51 | -0.33 | -5.13 | -8.75 | -26.23 | 5.88 | -34.78 | 6.75 | -10.71 | 17.21 | 5.99 | -5.52 | -25.33 |
| MCR2689C | Lymphoma | 0.17 | 5.21 | -30.03 | -21.33 | 19.47 | 1.85 | -18.13 | 18.46 | 36.85 | | -20.29 | -10.31 | 19.27 | -9.00 | -16.39 | 3.59 | -0.97 | 14.25 | 16.17 | -12.72 | 33.56 | 1.45 | -32.34 |
| MCR0152C | Lymphoma | -6.95 | 3.39 | 5.54 | -3.78 | 15.40 | -4.71 | 9.44 | -5.32 | 23.13 | | 0.66 | -10.08 | -0.19 | -3.66 | -0.72 | 0.46 | 1.45 | -0.72 | -1.78 | -1.65 | -0.43 | -6.31 | 3.33 |
| MCR0364C | Lymphoma | -4.55 | 35.33 | -3.27 | -4.50 | -4.02 | -2.47 | 35.89 | -3.37 | -4.05 | | -4.35 | -1.09 | 38.09 | -3.73 | -5.31 | -34.31 | 10.18 | -11.98 | -3.21 | -2.18 | -4.44 | -0.84 | -16.86 |
| MCR1177C | Lymphoma | -0.27 | -0.79 | -1.23 | -1.50 | -1.08 | -0.38 | 0.48 | -1.29 | 10.96 | | -0.66 | -0.72 | 9.47 | -0.39 | 0.38 | -1.35 | 1.49 | -0.01 | -0.35 | -0.96 | -0.27 | 1.52 | 6.87 |
| MCR1661C | Lymphoma | -0.56 | 3.78 | 1.14 | -1.90 | 4.48 | -2.42 | 2.30 | -0.74 | -0.60 | | 0.33 | 0.73 | 4.92 | -4.65 | 0.86 | -1.72 | 0.15 | -0.76 | 1.41 | -0.27 | -0.60 | -1.25 | 0.87 |
| MCR2106C | Lymphoma | -7.91 | 5.87 | -1.60 | -7.72 | 2.94 | -5.43 | 1.55 | -11.25 | 0.58 | | -2.76 | -3.19 | 15.58 | -10.49 | 0.79 | 9.32 | -2.92 | 2.67 | -0.59 | 6.07 | 8.59 | -3.97 | -15.06 |
| MCR1532C | Lymphoma | 1.85 | -0.50 | 1.55 | -9.75 | -0.79 | -11.40 | 0.07 | 14.45 | 15.80 | | -9.77 | -10.07 | 23.70 | -8.42 | 7.34 | 0.03 | 7.93 | -1.02 | -2.97 | 2.37 | -4.69 | -6.28 | -37.30 |
| MCR4966C | Lymphoma | -0.71 | 6.42 | -0.69 | -27.34 | 11.34 | 0.31 | 0.29 | 5.16 | 6.28 | | 0.84 | 20.32 | 1.53 | 1.35 | -3.54 | -26.02 | -7.49 | -8.55 | -8.02 | 0.42 | 3.70 | -0.85 | 0.72 |

**Supplementary Table6. The NPCC values and PTMs values of maternal liver cancer, breast cancer and lymphoma cases in the random forest classifier**

| Case ID | NPCC.BRCA | NPCC.LIHC | NPCC.DLBC | CA153 | AFP | CEA | CA199 | CA125 | CA724 | CYFRA211 |
| --- | --- | --- | --- | --- | --- | --- | --- | --- | --- | --- |
| MCR2809C | 0.37 | 0.33 | 0.31 | 13.21 | 141.40 | 10.21 | 19.77 | 18.22 | 3.35 | 2.01 |
| MCR2598C | 0.34 | 0.36 | 0.30 | 19.97 | 158.80 | 1.93 | 6.28 | 10.85 | 0.80 | 2.95 |
| MCR1266C | 0.35 | 0.35 | 0.30 | 11.47 | 44.47 | 0.96 | 3053.00 | 55.22 | 1.65 | 1.58 |
| MCR0336C | 0.38 | 0.34 | 0.27 | 8.51 | 157.50 | 0.75 | 11.81 | 147.80 | 2.27 | 3.23 |
| MCR1189C | 0.36 | 0.34 | 0.30 | 3.89 | 27.56 | 15.51 | 10.62 | 7.29 | 0.10 | 3.23 |
| MCR1260C | 0.35 | 0.34 | 0.31 | 23.75 | 38.48 | 1.65 | 56.17 | 40.45 | 0.17 | 1.18 |
| MCR1804C | 0.35 | 0.34 | 0.31 | 12.82 | 104.67 | 1.45 | 6.35 | 19.33 | 2.25 | 2.98 |
| MCR1105C | 0.37 | 0.35 | 0.29 | 300.00 | 57.15 | 63.20 | 241.02 | 41.45 | 0.25 | 3.24 |
| MCR2693C | 0.37 | 0.33 | 0.29 | 56.80 | 9.40 | 0.40 | 4.70 | 36.10 | 0.96 | 3.52 |
| MCR1124C | 0.35 | 0.35 | 0.30 | 18.90 | 149.70 | 0.72 | 15.62 | 540.82 | 0.55 | 4.42 |
| MCR4803C | 0.36 | 0.34 | 0.30 | 775.10 | 34.05 | 27.70 | 13.90 | 6.44 | 1.07 | 2.93 |
| MCR3067C | 0.35 | 0.35 | 0.30 | 377.50 | 91.95 | 7.66 | 22.62 | 121.40 | 3.55 | 24.21 |
| MCR2348C | 0.37 | 0.33 | 0.30 | 4.86 | 35.00 | 9.97 | 3.44 | 38.28 | 126.70 | 21.07 |
| MCR4035C | 0.34 | 0.35 | 0.31 | 16.38 | 90.29 | 0.55 | 9.47 | 113.80 | 1.80 | 0.36 |
| MCR6800C | 0.36 | 0.33 | 0.31 | 4.94 | 50.59 | 7.49 | 1279.00 | 161.40 | 1.82 | 9.39 |
| MCR0284C | 0.35 | 0.34 | 0.31 | 13.43 | 157.90 | 2.79 | 7.48 | 3.08 | 1.43 | 1.20 |
| MCR1448C | 0.35 | 0.34 | 0.32 | 56.30 | 1585.89 | 0.56 | 71.50 | 128.80 | 19.77 | 3.52 |
| MCR1661C | 0.34 | 0.35 | 0.31 | 10.78 | 1825.23 | 1.71 | 13.70 | 13.88 | 1.65 | 0.54 |
| MCR1669C | 0.39 | 0.33 | 0.28 | 18.28 | 1156.00 | 1.42 | 55.80 | 0.63 | 0.87 | 3.12 |
| MCR1361C | 0.34 | 0.32 | 0.33 | 18.72 | 240.00 | 1.02 | 19.54 | 16.69 | 1.02 | 11.70 |
| MCR1254C | 0.33 | 0.36 | 0.31 | 17.96 | 240.00 | 1.26 | 7.95 | 5.42 | 4.59 | 2.38 |
| MCR1327C | 0.33 | 0.36 | 0.31 | 1.89 | 220.05 | 0.70 | 11.26 | 14.96 | 4.25 | 2.15 |
| MCR1049C | 0.36 | 0.35 | 0.29 | 13.55 | 296.38 | 1.27 | 11.25 | 79.85 | 1.61 | 0.52 |
| MCR1376C | 0.34 | 0.35 | 0.30 | 13.35 | 240.00 | 1.20 | 2.10 | 16.13 | 3.42 | 1.52 |
| MCR2387C | 0.36 | 0.34 | 0.30 | 27.81 | 64.39 | 413.53 | 760.30 | 3.47 | 16.13 | 13.06 |
| MCR3313C | 0.36 | 0.33 | 0.31 | 11.02 | 110.80 | 4.71 | 110.90 | 8.10 | 551.00 | 2.26 |
| MCR6672C | 0.35 | 0.33 | 0.32 | 15.52 | 8630.35 | 1.89 | 2.48 | 11.27 | 3.56 | 0.24 |
| MCR2689C | 0.36 | 0.35 | 0.29 | 5.91 | 26.16 | 0.94 | 8.53 | 37.71 | 0.30 | 0.73 |
| MCR0152C | 0.36 | 0.33 | 0.31 | 17.00 | 34.60 | 0.50 | 7.20 | 10.00 | 7.82 | 3.52 |
| MCR0364C | 0.33 | 0.38 | 0.29 | 8.70 | 77.70 | 1.02 | 29.33 | 12.57 | 4.07 | 2.45 |
| MCR1177C | 0.36 | 0.33 | 0.31 | 1.50 | 3.02 | 0.42 | 2.51 | 3.35 | 2.37 | 1.95 |
| MCR1661C | 0.34 | 0.36 | 0.30 | 12.80 | 55.37 | 1.39 | 3.64 | 16.69 | 5.13 | 2.42 |
| MCR2106C | 0.34 | 0.36 | 0.30 | 11.48 | 50.08 | 0.31 | 6.45 | 21.25 | 0.24 | 0.48 |
| MCR1532C | 0.36 | 0.33 | 0.31 | 14.00 | 24.10 | 0.30 | 5.50 | 15.20 | 5.46 | 3.52 |
| MCR4966C | 0.34 | 0.35 | 0.30 | 0.40 | 202.32 | 1.60 | 33.36 | 8.70 | 13.14 | 6.82 |

**
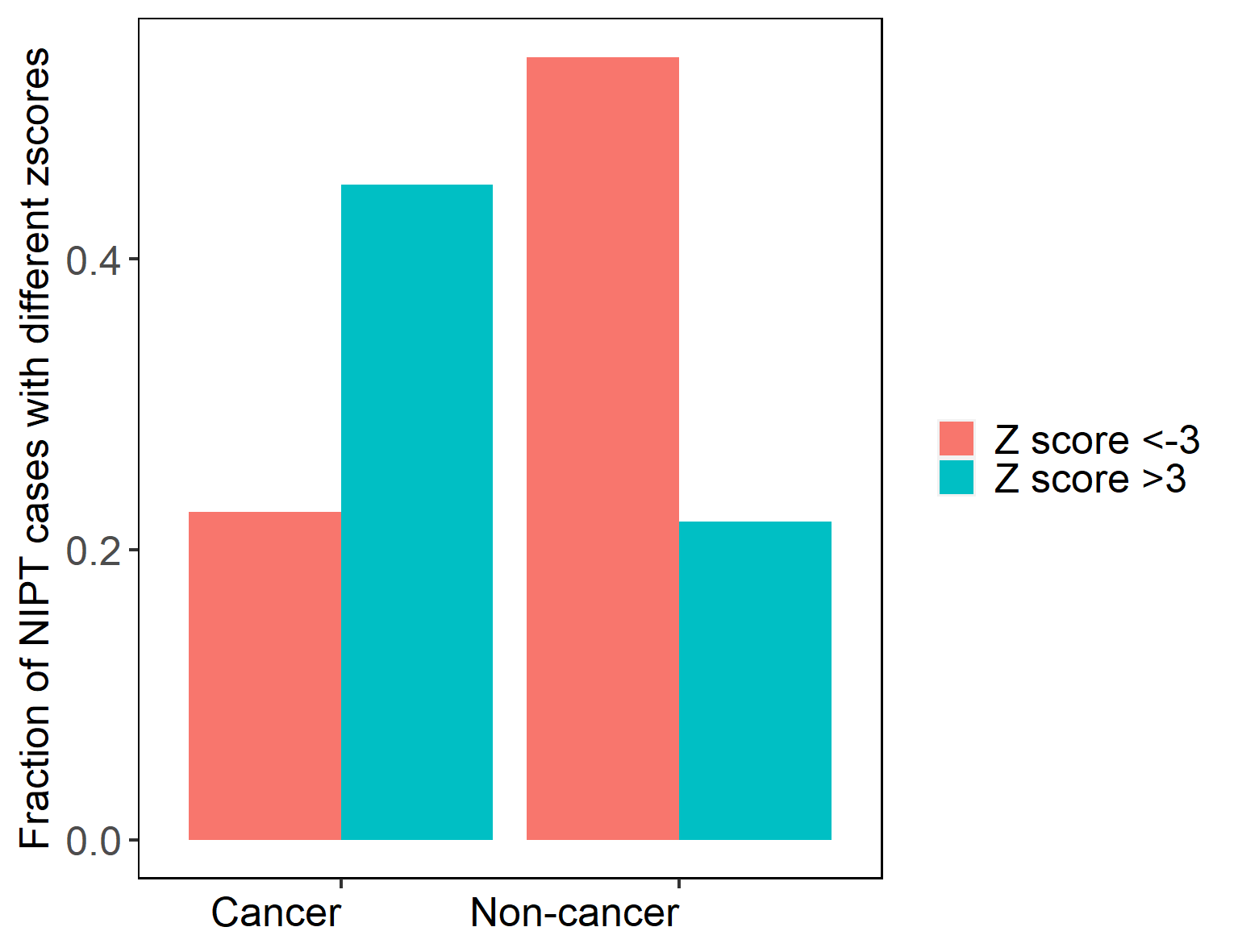
**

Supplementary Figure1. The fraction of NIPT tests showing significant amplification (Z score >3) and deletion (Z score < -3) of chr19 in non-Cancer and Cancer participants.


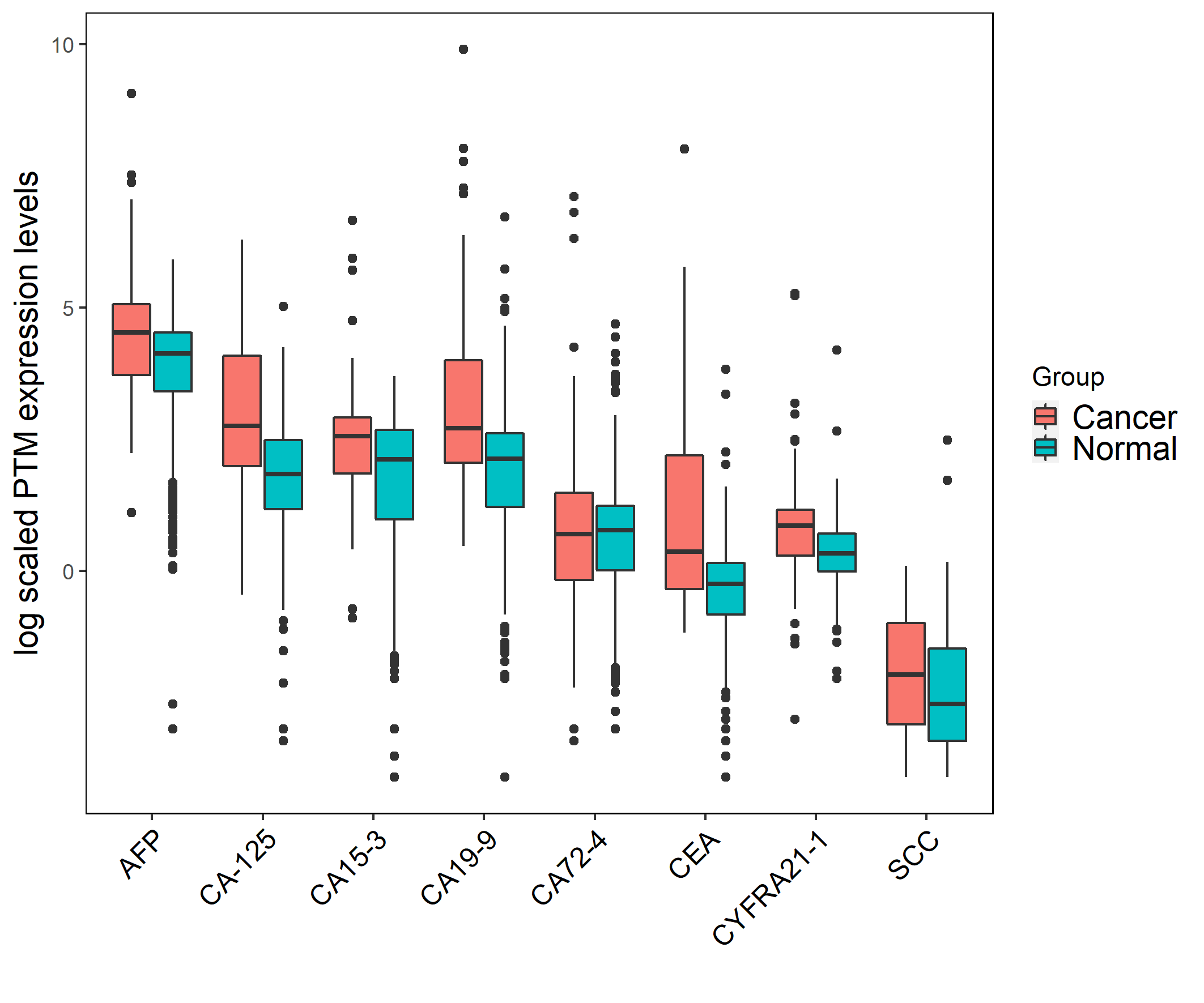


Supplementary Figure2. The difference in PTMs expression levels between cancer (n=56) and normal participants (n=451)


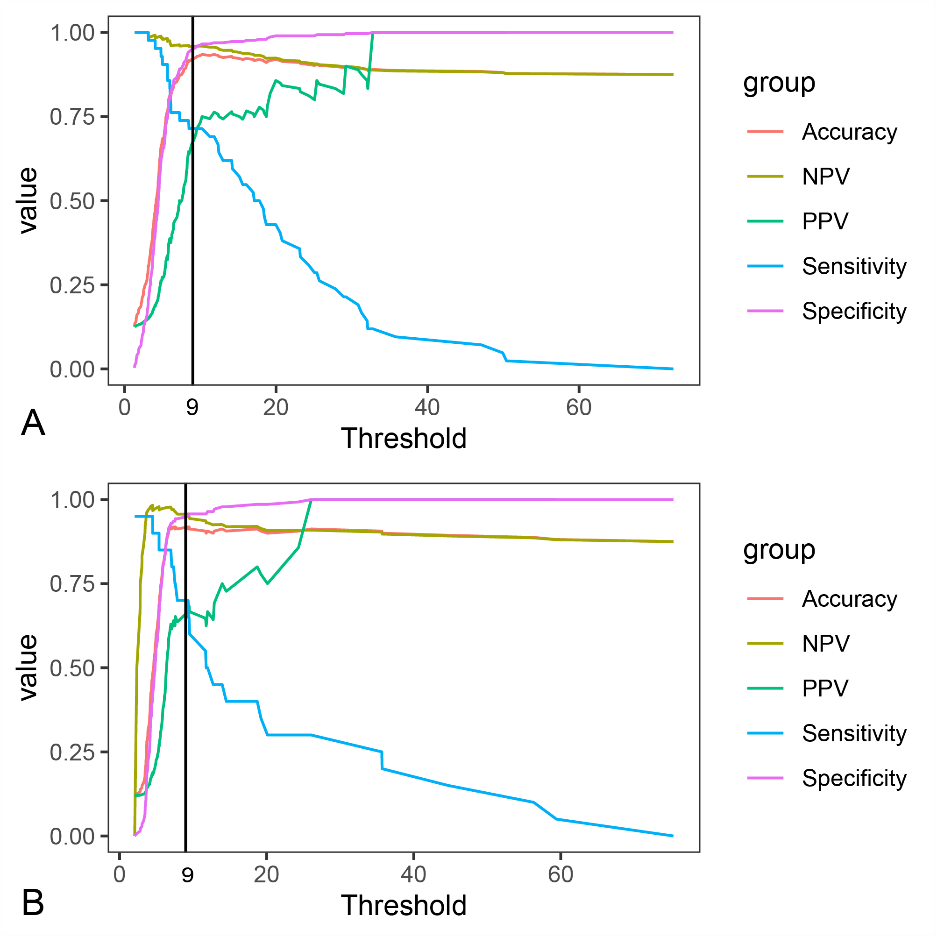


Supplementary Figure3. The performances of MTOP5Zscores in the identification of maternal cancer in the training and validation sets. A. The performances for MTOP5Zscores in the training set. B. The performances for MTOP5Zscores in the validation set. Notably, the vertical line indicates the cutoff to define women at high risk for maternal cancer.
